# 1315. Clinical, Radiological and Serologic Characteristics of Patients with Subarachnoid Neurocysticercosis: A Large Series from a Single Center in New York City

**DOI:** 10.1093/ofid/ofad500.1154

**Published:** 2023-11-27

**Authors:** Cesar G Berto, Robert Sacchi, Alexander Levitt, Elise O’Connell, Christina Coyle

**Affiliations:** Mass General Brigham, Boston, MA; Jacobi Medical Center - Albert Einstein College of Medicine, New York, New York; Albert Einstein College of Medicine, New York, New York; National Institute of Allergy and Infectious Diseases, Bethesda, Maryland; Albert Einstein College of Medicine, New York, New York

## Abstract

**Background:**

Subarachnoid neurocysticercosis (SANCC) is the most severe and difficult-to-treat form of neurocysticercosis (NCC).

**Methods:**

We describe the clinical, radiologic, and serologic characteristics of the largest series of patients with SANCC in a non-endemic area by reviewing the medical records of patients seen at Jacobi Medical Center in New York.

**Results:**

From 313 cases of NCC, 103 patients (32.9%) had subarachnoid involvement. Most individuals were male (65.1%) and born in Mexico (37.9%); the mean age was 41.4 years. Thirty-seven patients had only subarachnoid involvement, 50 patients had both parenchymal and SANCC, 5 patients had both intraventricular and SANCC, and 11 patients had all three spaces involved. Seventy-one patients (68.9%) presented with headaches. Of these, 35 patients had intracranial hypertension, and 27 required shunt placement. Thirty patients (29.1%) presented with seizures, most of whom had concomitant parenchymal disease. Fourteen patients (13.6%) presented as an ischemic event, 12 patients (11.7%) had symptoms due to spinal disease, and 8 patients (7.8%) had aseptic meningitis. Screening for spinal disease was performed on 61 patients. Of these, 14 patients (23.0%) had asymptomatic spinal involvement. Delay in diagnosis was found in 48 patients (49.0%) with a median of 12 months; chronic headache was the most common missed symptom (48.2%). Calcified SANCC was found in 37 patients (35.9%). Of these, 18 patients (18.4%) did not receive previous antiparasitic. Based on both CT scans and MRIs, 14 of these patients were categorized as completely calcified, and 23 patients were partially calcified with a cystic component seen on MR T2 images. The cysticercosis antigen in serum and CSF were negative in all cases of completely calcified and only in one partially calcified SANCC.

**Conclusion:**

This is the largest series of SANCC reported from a single center in the US and highlights the clinical spectrum. Our data also shows an important under-recognition of this severe form by the clinician and reports that calcified SANCC is common and can occur before and after treatment.

**Disclosures:**

**All Authors**: No reported disclosures

